# COVID-19: mobilising CT as a frontline management tool

**DOI:** 10.1259/bjr.20200522

**Published:** 2020-11-23

**Authors:** Imran Syed, Sami Khan, Tahir Khan, Sabeeh Syed, Taha Khan, Azhar Ali, Katherine Harries, Ermanno Capuano, Alexia Farrugia, Marcus Pittman, Godwin Simon, Tayyab Haider, Fawad Ali, Indrajit Gupta, Qaiser Malik

**Affiliations:** 1 Department of Radiology, Basildon and Thurrock University Hospital NHS Foundation Trust, Basildon Hospital, Basildon, UK; 2 School of Clinical Medicine, University of Cambridge, Cambridge, UK; 3 Acute Medicine, University Hospitals Plymouth NHS Trust, Derriford Hospital, Plymouth, UK; 4 Respiratory Medicine, Basildon and Thurrock University Hospital NHS Foundation Trust, Basildon Hospital, Basildon, UK; 5 Acute Medicine, Basildon and Thurrock University Hospital NHS Foundation Trust, Basildon Hospital, Basildon, UK; 6 Older People Medicine, Basildon and Thurrock University Hospital NHS Foundation Trust, Basildon Hospital, Basildon, UK

## Abstract

As the COVID-19 pandemic has spread across the globe, questions have arisen about the approach healthcare systems should adopt in order to optimally manage patient influx. With a focus on the impact of COVID-19 on the NHS, we describe the frontline experience of a severely affected hospital in close proximity to London. We highlight a protocol-driven approach, incorporating the use of CT in the rapid triage, assessment and cohorting of patients, in an environment where there was a lack of readily available, onsite RT-PCR testing facilities. Furthermore, the effects of the protocol on the effective streamlining of patient flow within the hospital are discussed, as are the resultant improvements in clinical management decisions within the acute care service. This model may help other healthcare systems in managing this pandemic whilst assessing their own needs and resources.

## Background

In March 2020, as hospitals throughout the UK prepared for an exponential surge in cases of coronavirus disease 2019 (COVID-19), it was clear that correct triaging and isolation of inpatients into specific wards was essential for optimal clinical management and infection control. A rapid and accurate diagnostic tool is vital for this purpose. Current practice advocates the use of real-time reverse transcription–polymerase chain reaction (RT-PCR) testing to diagnose COVID-19. Despite Public Health England efforts to provide faster RT-PCR testing capability, many hospitals lacked local testing facilities, with complete reliance upon stretched off-site providers.^1^ In addition, the literature cites that false-negative RT-PCR results are up to 29%.^2^ Timely and accurate ward allocation of suspected patients is essential to prevent cross-infection, as evidenced by a study in Wuhan which found that 41% of hospitalised COVID-19 patients were likely to have been infected during their admission.^3^ Therefore, the need for additional frontline investigations to be used alongside RT-PCR was highlighted, emphasising the value of imaging as a diagnostic tool in COVID-19.

In the clinical context of a high pre-test probability, numerous studies have reported CT scanning of the chest to be more sensitive than initial RT-PCR for the diagnosis of COVID-19. Fang et al^2^ found a higher sensitivity of CT chest (98%) when compared to that of initial RT-PCR (71%), whilst Ai et al^4^ reported that 97% of patients with RT-PCR confirmed COVID-19 showed positive findings on CT chest. Furthermore, a recent study from Italy analysed 158 patients and found the sensitivity of CT chest for COVID-19 to be 97%.^5^ Likewise, a study of 192 patients in Belgium reported a CT sensitivity of 87%.^6^ However, the reported specificity of CT scanning for diagnosing COVID-19 has varied between 25%, 54% and 94%.^5–7^ Therefore, it must be emphasised that only a positive RT-PCR result can confirm a diagnosis of COVID-19. Nevertheless, in the absence of swift testing capability, fever clinics in China used CT scanning as a triage tool, highlighting its role in the rapid detection of COVID-19.^7^

The revised British Society of Thoracic Imaging (BSTI) guidance on COVID-19, issued 16th March, recognised a diagnostic role for CT in the absence of RT-PCR testing availability, as well as acknowledging its potential to guide individual patient management decisions.^8^ This guidance also included a radiology decision tool which incorporated CT chest into the workup of COVID-19 patients. National Health Service (NHS) England has endorsed this decision tool by including it in their “Clinical guide for the management of radiology patients during the coronavirus pandemic”.^9^ In a later statement, the BSTI also advised that, in the context of a lack of initial RT-PCR testing availability and a normal chest radiograph (CXR), CT chest could be viewed as the “optimum initial diagnostic tool”.^10^

The role of CT in the management of COVID-19 was further recognised by a multinational consensus statement from the Fleischner Society, which was authored by a multidisciplinary panel of 29 clinicians.^11^ While acknowledging the superior capability of CT in the diagnosis of early parenchymal lung disease, the panel chose to leave the choice of imaging modality to clinical teams, based on their local resources. They concluded that in a resource-constrained setting, “imaging is indicated for medical triage of patients with suspected COVID-19 who present with moderate-severe clinical features and a high pre-test probability of disease”.^11^ Following this, the Radiological Society of North America COVID-19 Task Force issued best practice guidance incorporating the Fleischner Society’s statement regarding the use of chest imaging.^12^ An extended role of CT in diagnostic algorithms for COVID-19 patients was further highlighted in a recent review paper by Koo et al^13^, endorsing the guidance from the Fleischner Society.

While other hospitals have incorporated CT in a limited manner within their diagnostic algorithms for managing COVID-19 in patients,^14^ our protocol was first in the UK to utilise this modality as a frontline, point-of-care triage tool in the emergency department (ED). Our aim was to prevent the admixing of patients with varying probabilities of COVID-19.

## The Basildon protocol

**Figure 1.**
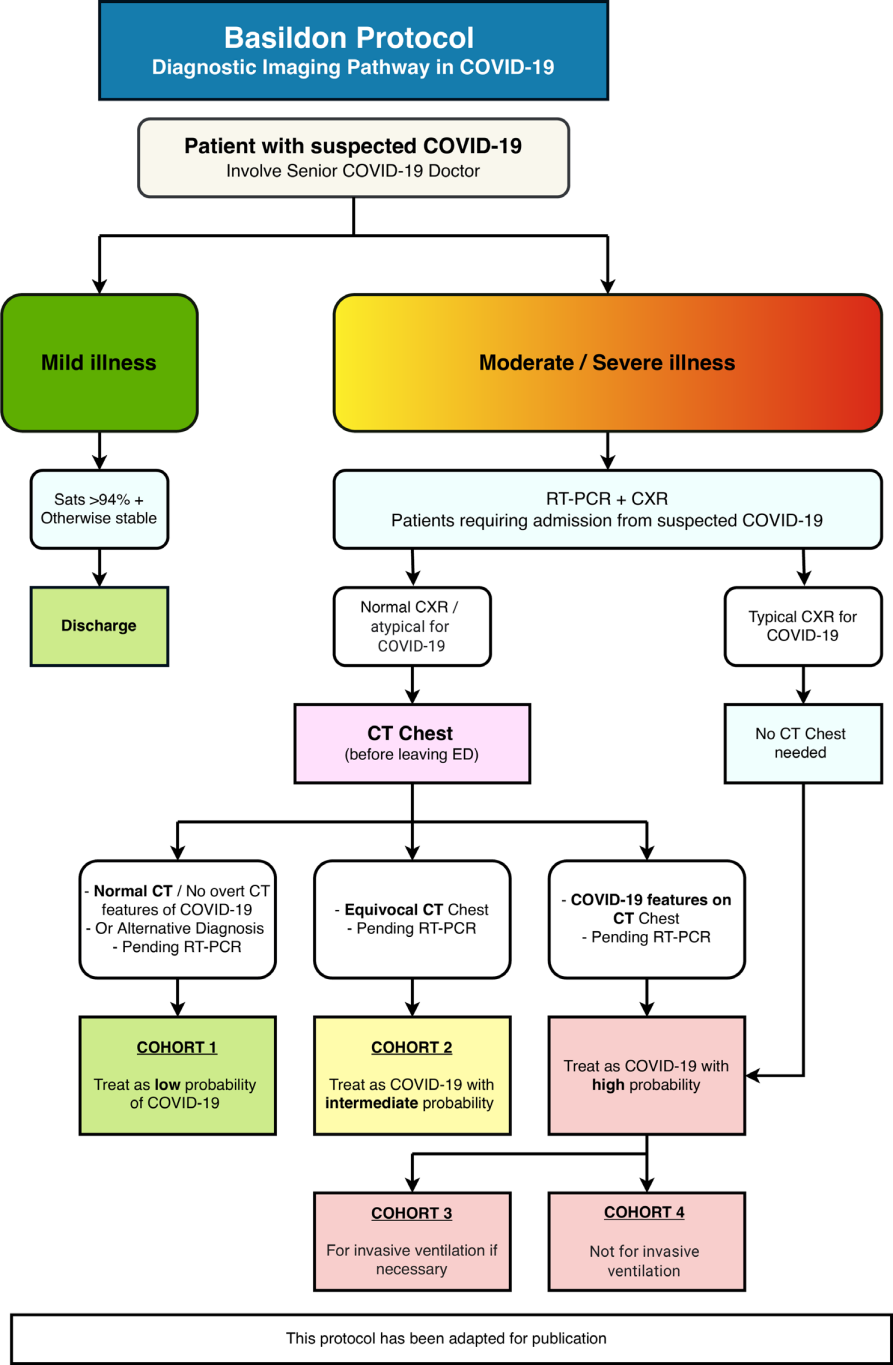
A flowchart of the Basildon Protocol-Diagnostic Imaging Pathway in COVID-19.

As COVID-19 has spread across the UK, Basildon University Hospital, a 712-bed institution located in close proximity to London, has been one of the most severely affected hospitals in the East of England. The high prevalence of cases within the region required preparation for a large influx of admissions. The major limitation in our hospital’s response was its reliance on off-site RT-PCR testing facilities, which delivered turnaround times of up to six days. This necessitated the use of CT to allow triage and cohorting of patients as they presented to the ED. Therefore, in accordance with the BSTI/NHS England decision tool,^9^ an algorithm (Figure 1) was created by a consortium of clinicians, including radiology, respiratory and acute medicine consultants.

Our protocol was initiated with an assessment of suspected COVID-19 patients by a senior clinician in the ED. These patients were then risk stratified into two categories: a stable group that was discharged (mild illness) and a group that required admission (moderate/severe illness). Mild illness was defined by an oxygen saturation above 94% on room air and clinical stability, while moderate/severe illness was based on senior clinical judgement, taking into account the clinical and biochemical parameters.

Admitted patients had a RT-PCR swab and a CXR in the ED. The diagnostic reporting of CXRs was based upon the BSTI’s guidance, resulting in four different codes; Classic/Probable COVID-19, Normal, Non-COVID-19 or Indeterminate for COVID-19.^15^ Those patients coded as Classic/Probable COVID-19 did not require further imaging and were treated as high probability of disease. The remaining patients required CT chest to determine their probability of COVID-19. Our CT scanner was available 24 hours a day and managed by specified staff with strict cleaning protocols post scan to ensure a streamlined service. The average time for a final authorised report on the Radiology Information System was 1.5 hours. The initial CT interpretation was performed by a general consultant radiologist, and a specialist chest radiologist was consulted for any abnormal chest CT findings prior to report authorisation. The real-time nature of this dual reporting system ensured that there was no impact upon cohorting. In conjunction with the BSTI’s guidance,^8^ CT features were used to determine the probability of COVID-19. Although the severity of disease on CT was reported, this did not influence patient cohorting as only the pattern of distributions of abnormalities on CT was relevant to determining the probability of COVID-19.

Based on imaging and a clinical assessment, each patient was placed in a cohort and allocated to the appropriate ward. Patients with CTs coded as Normal or Non-COVID-19 were determined to have a low probability of disease and thus placed in Cohort 1, while patients with CT scans that were indeterminate for COVID-19 were placed in Cohort 2 with an intermediate probability of illness. Patients with CTs or CXRs that were categorised as Classic/Probable COVID-19 had a high probability of disease and were thus placed in Cohort 3 or Cohort 4. These cohorts were separated, not by radiological parameters, but based on a clinical assessment of the potential to benefit from invasive ventilation. This focused on a cumulative weighting of the Clinical Frailty Scale,^16^ the presence of significant comorbidities and the patient’s clinical picture. The cohorting outcome always involved a senior respiratory physician in the decision process. Cohort 3 were considered appropriate for treatment escalation, whereas Cohort 4 were deemed to not benefit from invasive ventilation.

With this protocol in effect, during the month of April 2020, 43% of patients with suspected COVID-19 were deemed to have moderate/severe illness requiring admission. Of these, 12, 25, 33 and 29% were assigned to Cohorts 1 to 4, respectively. Notably, 48% of admitted patients had CXRs coded as Classic/Probable COVID-19 and subsequently did not require a CT chest on admission, reserving scanning resources. In order to accommodate the additional workload, our institution utilised one dedicated COVID-19 CT scanner, with the capacity to enlist an additional scanner if required.

The introduction of CT chest within the Basildon Protocol had a major impact on patient management. First, patients were transferred only once from the ED, through radiology, to a ward corresponding to the appropriate cohort, thereby limiting unnecessary exposure. Second, as COVID-19 cases increased, it was no longer possible to isolate all suspected patients in individual side rooms within our hospital. As a result, cohorting into designated wards enabled the categorisation of patients according to disease probability. These measures aided in minimising the risk of cross-infection for both patients and healthcare workers, while awaiting RT-PCR results. A further advantage of cohorting patients was in relation to the distribution of workload among staff. This was achieved by allocating different specialities to each cohort; Cohort 1 wards were staffed by general physicians, Cohorts 2 and 3 were managed by the respiratory team, and Cohort 4 was managed by the care of the elderly physicians.

## Conclusion

The Basildon Protocol highlights the pivotal role of CT in the workup of COVID-19 patients for the rapid assessment, triage and cohorting of patients in a resource-constrained environment with limited RT-PCR testing. These measures aim to reduce the risk of cross-infection between patients and healthcare workers and aid effective resource allocation. Using a designated CT scanner, specified staff, clear transfer policies and rapid reporting, the protocol was implemented efficiently and serves as a valuable management tool.
